# Recommendations for the transition of patients with ADHD from child to adult healthcare services: a consensus statement from the UK adult ADHD network

**DOI:** 10.1186/s12888-016-1013-4

**Published:** 2016-08-26

**Authors:** Susan Young, Marios Adamou, Philip Asherson, David Coghill, Bill Colley, Gisli Gudjonsson, Chris Hollis, Jane McCarthy, Ulrich Müller, Moli Paul, Mark Pitts, Muhammad Arif

**Affiliations:** 1 Centre for Mental Health, Imperial College London, Broadmoor Hospital, WLMHT, London, UK; 2 School of Human and Health Sciences, University of Huddersfield, Queensgate, Huddersfield UK; 3 Institute of Psychiatry, Psychology & Neuroscience (IoPPN), King’s College London (KCL), London, UK; 4 University of Dundee, Dundee, UK; 5 Education and Children’s Services, Perth, UK; 6 Division of Psychiatry and Applied Psychology, Institute of Mental Health, University of Nottingham, Nottingham, UK; 7 Cambridge University, Cambridge, UK; 8 Warwick Medical School, Coventry, UK; 9 South London and Maudsley NHS Foundation Trust, London, UK; 10 Leicester Adult ADHD Service, Leicester, UK

## Abstract

The aim of this consensus statement was to discuss transition of patients with ADHD from child to adult healthcare services, and formulate recommendations to facilitate successful transition. An expert workshop was convened in June 2012 by the UK Adult ADHD Network (UKAAN), attended by a multidisciplinary team of mental health professionals, allied professionals and patients. It was concluded that transitions must be planned through joint meetings involving referring/receiving services, patients and their families. Negotiation may be required to balance parental desire for continued involvement in their child’s care, and the child’s growing autonomy. Clear transition protocols can maintain standards of care, detailing relevant timeframes, responsibilities of agencies and preparing contingencies. Transition should be viewed as a process not an event, and should normally occur by the age of 18, however flexibility is required to accommodate individual needs. Transition is often poorly experienced, and adherence to clear recommendations is necessary to ensure effective transition and prevent drop-out from services.

## Background

Transition can be defined as *‘the process of change from one stage (state) to another stage (state)’.* There are many transitions that an individual makes throughout life, be they through *psychosocial* stages of development [7] or stages of cognitive development [22], and perhaps the most important transition is from adolescence to adulthood. This is a period of immense significance both in social and psychological terms, as well as in legal terms (i.e. legally becoming an adult at age 18). This is a period where young people are likely to move away from home, either to go to college/university or for job opportunities, start new relationships and assume new roles and responsibilities. This is also a period when many experiment in life, including alcohol and illicit substances. Any disruption during this period is likely to have longer lasting adverse effects on the young person’s development, realisation of their potential and possibly affect their psychological wellbeing.

Young people with mental health conditions are particularly vulnerable during the transition period, and disruption of care during transition adversely affects the health, wellbeing and potential of this vulnerable group [15, 25]. Poor transition leads to disruption in continuity of care, disengagement from services and is likely to lead to poor clinical outcomes [25]. Ideally, transition should be a planned, orderly and purposeful process, taking into account developmental and illness specific needs [24]. For the majority of patients transition is poorly planned, poorly executed and poorly experienced [25, 26].

### ADHD and transition

Attention deficit hyperactivity disorder (ADHD) is estimated to affect up to five per cent of school-age children and adolescents in the UK [20], with a peak incidence in those aged between six and 12 years [18]. Although ADHD was previously thought of as a childhood condition, meta-analysis of follow-up studies found that 15 % of children with ADHD continued to have clinical features that met formal diagnostic criteria at the age of 25 years, and a further 50 % continued to suffer significant impairment into adulthood [8, 31]. In addition, comorbid problems such as mood and anxiety disorders and substance misuse persist or develop in adulthood. Other associated problems that may beset adults include difficulties in interpersonal relationships, problems with further education, poor employment opportunities, a propensity for dangerous risk-taking behaviours, and high rates of offending [1, 32]. For a review of ADHD in adolescence, see [30]. This constellation of clinical problems and functional impairments emphasises the clear need for mental health services for adolescents and adults with ADHD. Yet data ascertained from 1,636 patients in the UK General Practitioner Research Database, containing data of over three million active patients between the eight year period 1999–2006, reported a great reduction in prescribing patterns during adolescence, in fact hardly any patients were receiving ADHD medication by age 21 [17]. Given the findings of Faraone et al. [8] and Cheung et al. [5], this drop in service utilisation cannot possibly reflect spontaneous remission of ADHD symptoms. Of greater concern is that it occurs at a time when young people need the support of these services the most, as adolescence is a risk period when mental health problems may become more complex and serious disorders emerge.

### NICE guidelines

In 2008, the National Institute for Clinical Health and Excellence (NICE) produced guidance about the recognition and management of ADHD across the life span. They emphasised that ADHD was a life-long condition and provided clear recommendations that care should continue from childhood to adulthood if symptoms persist. They also provided, for the first time in the UK, clear guidance for the development of transition arrangements from child to adult services:

1. Young people with ADHD who are receiving healthcare from child and adolescent mental health services (CAMHS) or paediatric services should normally be transferred to adult mental health services (AMHS) if they continue to have significant symptoms of ADHD or other coexisting conditions that require treatment.
2. Transition should be planned in advance by both the referring service and the receiving service. If needs are severe and/or complex, use of the Care Programme Approach (CPA) should be considered.
3. Patients should be reassessed at school-leaving age to establish the need for continuing treatment into adulthood. If treatment is necessary, arrangements should be made for a smooth transition to adult services, with details of the anticipated treatment and services that the young person will require made clear.
4. Precise timing of the transition arrangements may vary locally, but the transfer should usually be completed by the time the patient is 18 years of age.
5. During the transition to adult services, a formal meeting involving CAMHS and/or the paediatric service and AMHS should be considered, and full information about the adult service should be provided to the patient.
6. For young people aged 16 years and older, the CPA should be used as an aid to transfer between services. The young person, and when appropriate the parent/carer, should be involved in the planning.
7. After transition, a comprehensive patient assessment should be carried out. The assessment should look at personal, educational, occupational and social functioning, and it should evaluate any coexisting conditions, notably drug misuse, personality disorders, emotional problems and learning difficulties.
8. Trusts should ensure that specialist ADHD teams for children, young people and adults jointly develop age-appropriate training programmes for the diagnosis and management of ADHD.

### Common practice in the UK

Although publication of the NICE guidelines has raised awareness of the need for appropriate arrangements for the transfer of young people with ADHD from child to adult healthcare services, AMHS in the UK for ADHD remain patchy. Furthermore, there is significant variation in the ways that services are organized across the UK and it is likely that some of this variation impacts significantly on the effectiveness of transition between adolescent and adult services. Healthcare for children with ADHD is generally provided by either community paediatric services or by CAMHS, or sometimes a combination of the two, that may or may not work in a joined-up manner. The interface between these services and AMHS is often complex with the potential for significant gaps. A survey conducted by Hall et al. [13] reported the absence of joint working between CAMHS and AMHS to be 66 % and 59 % of child and adult services, respectively. Furthermore, all 24 respondents from adult services reported having no official transition policy in place, and 89 % of National Health Service (NHS) Mental Health Trusts reported an absence of dedicated staff to support the transition. Surveys of NHS Trusts have also indicated a nationwide lack of accurate data pertaining to both the number of adults with ADHD within individual trusts, and the number of children/adolescents transitioning to adult services [12].

There seems to be a difference in practice between community paediatric and child & adolescent mental health services. Traditionally, paediatric services have stopped providing care for medical problems early in adolescence, although most now continue to provide care until school-leaving age. In the past most CAMHS generally stopped providing care either at 16 years of age or at school-leaving age (whichever was later). However, there is now a shift towards CAMHS continuing to provide care for patients up to 18 years of age regardless of school status. It has been suggested that AMHS are often too rigid in their age criteria for transition, with reports of refusals to consider transition until the individual reaches the age of 18 [3]. To further complicate issues it is reported anecdotally that whatever the technical cut-off age may be, many paediatric and CAMHS teams continue to see young people well past this age, due to the difficulties in transferring care to adult services [33]. Hence there is a mismatch in the expectation about when care should be passed from adolescent to adult services. Such inconsistencies have been reported to be disruptive and unsettling for patients [28] and this disruption to continuing care is detrimental to overall clinical care from both an individual and service perspective.

### Expert workshop

In order to facilitate further discussion about the transition from child to adult services and develop more explicit and comprehensive recommendations for clinicians, commissioners and policy makers, an expert workshop – “ADHD: Transition from Adolescence to Adult” – was convened in June 2012 in London by the UK Adult ADHD Network (UKAAN) [29]. UKAAN is an organisation founded in 2009 by a group of mental health specialists in response both to the NICE guidelines [19] and to recommendations from the British Association for Psychopharmacology (BAP) [21] that aims to provide support, education, research and training for mental health professionals working with adults with ADHD. The workshop was attended by experts in the field of ADHD, working across services, together with allied professionals and patients. The workshop consisted of a series of presentations summarising the transition process from the perspective of these experts. The workshop first considered the findings of the TRACK study [27] which sought to identify factors that facilitate or impede effective transition more generally within the healthcare system. The National Institute for Health and Care Excellence,[19]) guidelines for transitioning young people with ADHD and the extension to these recommendations proposed by Young et al. [33] were reviewed, and consideration was given to the broader clinical, educational, occupational, and social needs of young people in this age group. There followed an in depth discussion tabling a consensus for detailed recommendations on the provision of transition services for patients with ADHD.

### Setting the Context – Findings from the TRACK Study

The expert workshop commenced with a summary of the findings from the Transitions of Care from CAMHS to AMHS (TRACK) study [27]. The specific aims of the TRACK study were to identify factors that impede or facilitate effective transitions and continuity of care across the mental health services for children and adolescents, covering all disorders and not limited to ADHD, and to make recommendations about the configuration of services.

Healthcare transitions are commonly defined in the literature as a purposeful, planned process that addresses the medical, psychosocial and educational or vocational needs of young people and young adults with chronic physical and medical conditions as they move from child centred to adult orientated healthcare systems. An important context for considering healthcare transition for young people with long-term health conditions is that they typically face several types of transition simultaneously – developmental transitions from childhood to adolescence to adulthood, situational transitions from child services to adult services, and possibly even transitions from relative health to illness or, indeed, sometimes in the other direction. As young people make these transitions they generally take on more responsibility from their parents/carers for all aspects of their healthcare, as well as for other areas of their life. As a consequence it is inevitable that, at the same time, parents/carers face a transition process of their own. Hence the transition from child to adult health services brings service-related challenges associated with an inherent shift from a family-orientated service to an individual-orientated service. Whilst the TRACK study focused on those transitioning between child and adult services, not all patients with ADHD will necessarily be in contact with child services at the time of transition. Indeed many of those with ADHD will not have received specific healthcare for their problems during childhood and adolescence – and may be presenting formally for the first time at transition from adolescence to adulthood.

The TRACK project was a multi-site mixed models study, undertaken in six mental health trusts within England. The key findings were:

- Whilst many of the identified transition protocols were based on policy documents, there was evidence of a policy-practice gap, as services differed considerably on their interpretation of both policy and the recommendations for the practical aspects of transition. Although most of the protocols identified the patient as central to the transition process, few, if any, specified ways of preparing them for it.
- Optimal transitions were defined as those demonstrating evidence of: information transfer across services; a period of joint working between CAMHS and AMHS; transition planning involving key professionals from both services, patients and/or their parent/carers; and continuity of care. Transitions were considered sub-optimal if they failed to meet one or more of these criteria. The main findings were that, whilst around 80 % of patients were considered suitable for transition, around one-third were not referred to adult services. Adult services accepted the vast majority of referrals, however of those accepted around 20 % were discharged without being seen and only four per cent experienced optimal transition.
- An organisational analysis showed that CAMHS were seen as being more person-centred, holistic and family orientated, and adult services as more medication focused and crisis orientated. Facilitators for transition included: dedicated transition posts, joint working, early communication and greater involvement of carers. Barriers for transition included variability of eligibility criteria, differing thresholds between AMHS and CAMHS, communication problems between services and lack of confidence amongst adult staff on managing young people in general, and specifically relating to those with neurodevelopmental disorders.
- In a qualitative study of patients’, carers’ and mental health professionals’ experiences of transitions, participants described the importance of transfer planning meetings, joint working between the two services, and good information transfer. Interestingly most young people preferred not having their parents/carers involved with their care at AMHS, and many said that they appreciated the break from this family orientated system because they wanted to be more autonomous. On the other hand parents/carers wanted greater involvement with adult services.

Despite making up a large proportion of the caseloads of CAMHS services, children with ADHD were relatively under-represented within the TRACK sample, making up just under 10 % of the total sample. Only six out of the 15 cases reviewed were transitioned to AMHS and none of these were classified as being ‘optimally transitioned’. Overall those young people with neurodevelopmental disorders such as ADHD and autism spectrum disorders, and those with emotional disorders or emerging personality disorders, were more likely to fall through the gaps. Those with serious mental illness, such as psychosis, patients who are receiving pharmacological treatment and with history of inpatient treatment are more likely to be accepted for transition.

### Patients’ perspectives

There followed representations from patients on their experience and knowledge of the transition process. The transition period is a time of changing responsibilities, in which the young person is assuming greater autonomy and increased responsibility for his or her own care. Nevertheless, it is important that parents/carers should feel that they are involved in the transition process. This can be challenging because young people do not always want their parents/carers to continue to be involved in their medical care or the management of their condition. However, AMHS need to be willing to work more closely with parents/carers and to recognise that ongoing parental support for the young person is often necessary. This of course can only be achieved with the patient’s permission.

It was stressed that transition should start much earlier than is generally recognised. There was a clear call for psychoeducational material for patients and families, as they described wanting to be informed much earlier in the process about their condition and what to expect as they move into adulthood. Specifically they asked for greater detail about their diagnosis and its prognosis, treatment options, involvement in decision making and a specified care plan. In addition, they wanted the opportunity to share with professionals their feelings and their desire to take control of managing their condition and play an active role in their care plan.

It was emphasised that transition needs to be seen as a “process” rather than an “event”. Specifically, young people need to know what can be expected of these services, what AMHS offer and what they do not offer. They thought that transition meetings attended by CAMHS and AMHS were a good idea as this would facilitate a smoother transition. The potential value of lifespan clinics in the management of ADHD was highlighted. Patients were mindful however of the support they need in this process due to organisation and planning deficits. Support groups play an important role in the process, providing support for the young person and their family; the latter for the first time have to take a step back with a less proactive role.

The experience described at the workshop strongly resonates with perspectives reported in the Transition Into Adult Mental Health Services (TRAMS) study [28]. This was a qualitative study of the experiences of 10 young people with ADHD, and their parents/carers, in transition from CAMHS to AMHS. Four key themes were identified from the analysis:

1. Clinician Qualities and Relationship: These were identified to be an integral component of patient satisfaction, with a particular focus on the need for continuity of listening throughout the transition process, non-judgemental support and a practical, solution-focused approach targeting achievable and realistic goals.
2. Responsibility of Care: A number of issues arose regarding the question of who is responsible for providing care and ensuring continuity of care. From the young person’s perspective, a conflict can emerge between the greater autonomy expected in AMHS and the difficulties they experience in areas such as planning and organisation, for which they typically rely on parent/carer support. Inevitably for parents/carers, there is a responsibility to balance support and autonomy. Ultimate responsibility for providing a consistent transition lay with clinicians, with the importance of joint meetings between CAMHS and AMHS, alongside patients and their families, as well as an overall clear plan for handover suggested as integral components of a seamless transition.
3. Nature and Severity of Problems: Acceptance by AMHS appeared to be contingent on the nature and severity of the young person’s problems. There was a suggestion that transition may go more smoothly for those presenting with more severe problems, such as self-harming behaviours, and higher levels of comorbidity. For those whose problems were well controlled at the time of transition, neither the AMHS nor the young person themselves recognised the need for the on-going support required to sustain this level of health.
4. Expectations of AMHS: The expectations of AMHS from patients and their families were often high, although many of these are unlikely to be met (i.e. assistance with housing). It is essential therefore that CAMHS clinicians do not provide unrealistic expectations of AMHS, with clear information offered prior to transition concerning exactly what can and cannot be offered.

### The transition process

Most AMHS are set up to provide care for patients with serious mental disorders such as psychosis. In the TRACK study, only one in five of the 17 year olds in CAMHS with neurodevelopmental disorders (including ADHD) made a successful transition to adult services [27]. This contrasts with the more successful transition of young people with psychosis and other conditions considered to be serious mental disorders. Moreover, experience suggested that patients with ADHD and comorbid mental health problems, such as depression and suicidal ideation, often have an easier transition to adult services than patients diagnosed with ADHD alone. Nevertheless, one-half of all patients fail to adhere to treatment guidelines [9] and discontinue treatment within 2–3 years of starting pharmacological therapy [11, 34]. Some common reasons for the discontinuation of treatment are: adverse effects, ineffectiveness/suboptimal response, poor adherence, patient/caregiver decision and symptom relief. Other less common, although important, reasons include a dislike of medication, dosing inconvenience and social stigma [10].

It needs to be emphasised that transition to adult care is a process, rather than an event. For their part, CAMHS should provide timely and accurate information to patients about adult services and develop specific mechanisms to overlap the transitional arrangements with adult services. The transition process can be used to prompt a review of what other organisations may be required in the patient’s care. Indeed, this is an opportunity to inform appropriate agencies and organisations, with the patient’s consent, of their current circumstances. For this to be achieved, a good working knowledge of the common partner organisations in the area is crucial. These may be statutory and/or local organisations, such as child protection services, the local authority (for assessment for social care), local educational facilities and resources, court diversion services, and youth offending teams. In addition, there are an increasing number of voluntary and third-sector agencies that provide help and support. It is also advisable to involve the patient’s general practitioner (GP) because often this is the most stable service provider during the period of transition.

It was proposed that for the transition to adult care to work well, adult psychiatry needs to accept referrals from CAMHS and community paediatric services, and develop a better understanding of their needs. This will involve targeted continuing professional education on ADHD and, in particular, on the transition process. Of concern is that there is no specific provision to ensure ongoing care for ADHD patients who are not accepted by AMHS. There followed discussions regarding the best model for service provision and, in particular, whether adult-focused neurodevelopmental services need to be specifically developed. These already exist in some parts of the UK and were considered useful (see Table 1). To avoid excessive resource implications and an unnecessary division to the process, these are best located as an extension of CAMHS with a set agenda that includes psychoeducation, goal setting, risk reduction, coping strategies, employment and education. Additional consultations can be added on an ‘as needed’ basis, e.g. with a pharmacist, social worker, psychiatrist, psychologist and/or a member of the youth offending team.

**Table 1:** Transition practice in Leicestershire

Leicestershire have developed a robust transition protocol ensuring that all referrals from children’s services are carefully reviewed and appropriate action is taken. In Leicestershire, referral acceptance by the adult ADHD service is 100 %. Children services are advised to start the referral well in advance of the point of transition on the patient’s 18th birthday. They are also advised *not to discharge* the patient before they have been seen by the adult service. This ensures that the patient is never lost between the two services. In case the patient does not attend the appointment arranged by the adult ADHD clinic, the referring clinician is contacted for further advice and efforts are made to encourage the patient to attend. When dealing with complex cases or difficult to engage patients, a period of joint working has also been found to be useful.
Recognising the vulnerability of patients through this period of transition and the need to ensure continuity of care and stability, a great degree of flexibility is exercised when dealing with this group of patients. Even when the patient is not keen on taking the medication or attending further reviews, they are not discharged from the service. Time is spent with patients providing psychoeducation about the disorder, its course and the consequences of untreated ADHD. During these discussions it is important to acknowledge the patient’s views and reservations, for example, stigma about a psychiatric diagnosis label, their wish for autonomy, their view that they can manage their symptoms without the help of medication etc. They are offered a further appointment and are encouraged to take their time before making a final decision. In the large majority of cases, patients decide in favour of continuity of care.

### ADHD, education and employment

We can be fairly confident that key transitions, and in particular, those bridging secondary education and the world of further learning, independent living and work, constitute a period of heightened vulnerability in the life of any young person with ADHD.

However, the absence of a reliable evidence-base to inform generalisations about the employment experience of young people transitioning from school or college to the world of work is complicated by the heterogeneity of the ADHD population in terms of cognitive ability, comorbidity, gender expression, and treatment uptake, as well as geographical variations in the labour market.

Whilst it is fair to suggest that young people with ADHD suit better those occupations that provide novelty, physical activity and immediate feedback, and which are thus non-repetitive, highly structured and lacking in organisational/time-management demands, the prospects for any individual are likely to hinge on a number of other factors which may be either directly or indirectly related to the way that ADHD has manifested itself during their formative years.

Sufficient anecdotal evidence exists to complement studies in USA and Europe to suggest that rates of economic activity and thus income levels are significantly lower for young adults with ADHD, and that such differentials often persist throughout the life span [4, 6, 16]. The treatment effect is poor for occupational outcomes [2], however there is an emerging body of data to support the positive impact of personalised assistance in negotiating the labour market by helping job-seekers with the application and interview process, providing structures in daily living to manage financial obligations and punctuality, and in averting negative tendencies towards substance misuse, social conflict and offending [14, 23]. Likewise, adjustments in the workplace, such as a re-framing of organisation demands or support with time-management, may allow workers with deficits in these areas to exploit relative strengths in others and thus provide a net gain to the employer [1]. Programmes to improve the employability of people with ADHD are likely to be most effective when they are sensitive to the current needs and future potential of each individual, multi-modal in delivery, and which address gaps in prior learning that impair recruitment prospects.

### Consensus

Having considered all the available evidence and expert opinion, the expert group deliberated the recommendations that need to be made to achieve a smooth and effective transition from child to adult services for young people with ADHD, and prevent drop out from services. The earlier recommendations of Young et al. [33] were used as a starting point and framework for discussion. When using these guidelines and recommendations, local situations will need to be considered when designing transition services as there is a considerable variation in service design and delivery between regions. Consensus was reached for general recommendations (Table 2) and specific recommendations (Table 3).

**Table 2:** General recommendations for transition of care from children’s to adult services

1. Clear transition protocols should be developed jointly by commissioners, CAMHS/paediatric services, AMHS, primary care, and other agencies as relevant to facilitate transition and ensure that standards of care are maintained during the transition period.
2. These protocols should specify timeframes, lines of responsibility, who should be involved, how the young person should be prepared, and what should happen if AMHS are not able to accept the referral.
3. Protocols should allow for flexibility in the age of transition so as to accommodate developmental needs and stages, but there should be explicit referral criteria and service provision. Ideally, transition should occur at a time of clinical stability. Patients should not have to relapse or have worsening mental health in order to continue to be able to access services.
4. Transition protocols should be available to all clinical teams and should include psychoeducational material that provides high-quality, comprehensive, impartial and appropriately written information for both young people and their parents and carers. There is a need for more age-appropriate psychoeducational material for patients at the transition stage. This material should include information about ways that young people can manage their own symptoms and problems, and access advice and support. Information should also be developed in a media format that is readily accessed by young people, e.g. use of phone applications and internet sites.
5. The needs and wishes of parents/carers should be respected and their ongoing involvement with the young person negotiated. Some parallel services that can provide information and support for parents/carers during the transition period may be required.
6. Efforts should be made to inform and educate allied professionals who may come into contact with young people with ADHD for the first time during the transition period, e.g. forensic medical examiners and those working in the probation services and in correctional units and prisons.
7. Healthcare jurisdictions should be encouraged to use similar care pathways and outcome measures across different patient age groups.

**Table 3:** Specific recommendations for transition of care from children’s to adult services

1. A planned transfer to an adult service should be made if the young person continues to have significant symptoms of ADHD or other co-existing conditions that require treatment
2. Transition should be planned well in advance by both referring and receiving services. Timings of transition may vary but should ordinarily be completed by the age of 18 years. Transition between teams should be a gradual process and should be thought of as a ‘process’ and not a ‘single event’.
3. Patients should be involved in discussion about transition and informed of the outcome of any transition assessment. The transition process should proceed according to need in terms of future medical care (e.g. involvement of general practitioner [GP] services, specialist adult ADHD teams, adult learning disability services, adult physical health teams). Importantly, the GP should be involved throughout the process.
4. Discussion, and where necessary, joint meetings between child and adult services must ensure that the needs of the young person will be appropriately met. It is important to consider the presence of comorbid and/or related problems, which may involve further discussion and collaboration with educational, or occupational and social agencies.
5. CAMHS practitioners and paediatricians should foster engagement with AMHS through open discussion and psychoeducation about ADHD, the benefit of evidenced-based psychological and pharmacological treatment where appropriate, and the risks of disengagement. It is important to address concerns about stigma associated with referral to AMHS.
6. For young people aged 16 years or over in CAMHS, a CPA should be used to aid transfer. CPAs are not available in paediatric practice, and so a planned assessment of need with the young person and their parents/carers and a clearly documented plan of action is recommended.
7. Parents/carers need to be prepared and facilitated to aid their child’s gradual move towards independence and autonomy (with respect to the management and treatment of their ADHD). The referring and receiving healthcare teams should be mindful of possible parental ADHD and when this is present (or suspected) provide appropriate support.
8. Shared care arrangements between primary and secondary care services for the prescription and monitoring of ADHD medications should be continued into adulthood.
9. Direct psychological treatment should be considered (individual and/or group Cognitive Behavioural Therapy) to support young people during key transitional stages. This should have a skills development focus and target a range of areas including ADHD symptoms, social skills, interpersonal relationship problems (with peers and family), problem solving, self-control, dealing with and expressing feelings. Active learning strategies should be used.
10. Specific protocols need to be developed for young people who are not accepted by AMHS criteria, but whom the referring service strongly believe need ongoing support. Care needs to be taken that these patients are not left without the support they need during this very important transition period.
11. Separate care pathways should be developed for young people who drop out of CAMHS or paediatric services when they are under 18 years of age, and who later re-present in the healthcare system as adults.
12. Separate care pathways should be developed for patients who come to the attention of the healthcare system on account of ADHD for the first time as adults.
13. The referral letter from children’s services should provide a comprehensive account of the patient, including: diagnostic summary and formulation; treatment history; rationale and response; side effects, compliance, abuse and diversion issues, and ongoing treatment needs; any psychiatric and medical comorbidities, their impact on ADHD and treatment; any other ongoing needs - social, financial, accommodation or occupational and an updated risk assessment.
14. The adult service should acknowledge the receipt of the referral. The patient should not be discharged by the children’s services until they have been seen by the adult services and their care has formally been taken over by the adult services. This provides a safety net and reduces the likelihood of patients dropping out of the services during the transition period.
15. Following acceptance of the referral, the adult service should allocate a key worker/lead clinician who will coordinate the care needed.
16. When dealing with patients who are anxious about the transfer of care to adult services or those with complex needs, it may be necessary for children’s services to joint work with adult services for a few months to facilitate the transfer of care.

### Future research

In the course of the workshop it became clear that little is known about the outcomes of individuals with ADHD who fall through care gaps, patients who remain under the care of Primary Care services throughout transition into adulthood, interventions that might improve the process, and patient and carer experiences. Thus recommendations were also made for future research into areas relevant to the transition of patients with ADHD from child to adult services, including:

1. The reasons why patients stop taking medication during the transition period.
2. The reasons why patients lose contact with their ADHD healthcare system during the transition period, including why adolescents chose to discontinue treatment and how to address issues of decreased efficacy of medication over time.
3. Pharmacological strategies to identify patients, before their transition to adult services, who are at risk of developing comorbid psychiatric difficulties, with a view to preventing the development of these comorbid problems.
4. The effectiveness of non-pharmacological interventions in this age group.
5. Females with ADHD are underrepresented in the research literature, and their position through the transition process demands more attention.

## Conclusions

Transition is a process, not an event, and normally occurs by the age of 18. However, flexibility is required to accommodate individual needs. Unfortunately, transition is often poorly planned, executed and experienced. Adherence to clear recommendations is necessary to ensure effective transition and prevent drop-out from services.
